# Renal osteodystrophy in the obesity era: Is metabolic syndrome relevant?

**DOI:** 10.1371/journal.pone.0180387

**Published:** 2017-07-18

**Authors:** Janaina Da Silva Martins, João Henrique Castro, Nestor A. Sainz Rueda, Luciene Machado dos Reis, Vanda Jorgetti, Rosa Maria Affonso Moysés, Jacqueline Teixeira Caramori

**Affiliations:** 1 Nephrology, Department of Internal Medicine, Faculdade de Medicina Botucatu Univ. Estadual Paulista-UNESP. Botucatu, Brazil; 2 Department of Medicine, Universidade Estadual de Maringa, Maringa, Brazil; 3 Multidisciplinary Clinical Nutrition Team, Universidade Estadual de Maringa, Maringa, Brazil; 4 Nephrology Division, Universidade de São Paulo, São Paulo, Brazil; University of Milan, ITALY

## Abstract

**Background:**

Observational studies have shown a beneficial effect of obesity on bone health; however, most of those studies were not based on bone biopsies. Metabolic syndrome (MetS) could have an effect on bone remodeling. However, there are no data on the effects of MetS in the presence of renal osteodystrophy.

**Objective:**

The aim of this study was to investigate associations between MetS and renal osteodystrophy using the bone histomorphometric turnover-mineralization-volume (TMV) classification.

**Design, setting, participants & measurements:**

This observational cross-sectional study included 55 hemodialysis patients (28 women/27 men) who were evaluated for MetS and bone histomorphometry. Biochemical parameters included calcium, phosphorus, alkaline phosphatase, intact parathyroid hormone (iPTH), 25-hydroxyvitamin D, free serum leptin, fibroblast growth factor 23 (FGF23), intact osteocalcin, sclerostin (Scl), glucose, insulin, and thyroid hormones. Robust models of multivariate linear regressions were used for the statistical analyses.

**Results:**

Females had higher iPTH levels (1,143 *vs*. 358, p = 0.02). Patients with normal bone volume (BV/TV) had a higher prevalence of MetS (73.6% *vs*. 41.7%, p = 0.02) and higher serum phosphorus, C-terminal FGF23 and insulin levels. The multivariate regression analysis showed that low-density lipoprotein cholesterol (LDL) was positively correlated with bone formation rate (BFR/BS) and negatively associated with mineralization lag time. Bone volume was negatively associated with age but positively associated with MetS. Body mass index (BMI) was not correlated with any of the bone histomorphometric parameters.

**Conclusion:**

Our results suggest that MetS is not a risk factor for low bone volume in hemodialysis patients. Furthermore, BMI alone was not related to bone volume in this population.

## Introduction

The prevalence of obesity more than doubled between 1980 and 2014, and this condition affects 600 million adults globally, which corresponds to 13% of the world’s adult population (11% of men and 15% of women) [1]. Although obesity is associated with a number of comorbidities, such as metabolic syndrome (MetS) and chronic kidney disease (CKD) [2], obese patients have historically demonstrated advantages in terms of bone density, especially in the hips and femurs [3,4].

The potential mechanisms through which obesity and/or MetS act on the skeleton have been the focus of several studies with inconsistent results. Among patients with CKD, the controversy is even greater, and the data are often confounded by bone and mineral disorders related to CKD (CKD-MBD). The fracture risk in hemodialysis patients is double that in the normal population, and the mortality of these patients is higher than in patients without fractures, regardless of age, duration of dialysis or cardiovascular disease [5]. In this population, a bone biopsy is the gold standard method for evaluating bone volume, turnover and mineralization [6].

The number of obese patients receiving hemodialysis has increased rapidly among the global population, and in the United States, approximately 35% of patients waiting for a kidney transplant have a body mass index (BMI) higher than 30 [7]. Obesity has been associated with longer survival in hemodialysis patients [8], and there are no data from bone biopsies to determine the association between obesity and MetS with CKD-MBD.

Thus, the aim of this study was to identify the relationship between obesity and MetS with CKD-MBD. In addition, the associations of leptin, osteocalcin (OC), fibroblast growth factor 23 (FGF23), sclerostin (Scl) and insulin resistance index (HOMA-IR) with bone biopsy results were investigated.

## Materials and methods

### Subjects and study design

This was a cross-sectional, observational, prospective and non-randomized study that included hemodialysis patients from the Hospital da Faculdade de Medicina de Botucatu, Universidade Estadual Paulista (UNESP). All patients were older than 18 years, had received hemodialysis three times per week for at least six months and agreed to a bone biopsy. The exclusion criteria were as follows: presence of cancer or advanced liver disease and use of glucocorticoids, immunomodulators or antiretroviral drugs in the past six months. Anthropometric analyses, blood samples and bone biopsies were obtained at the same time. Demographic and clinical data, such as age, race, gender, baseline renal disease, comorbidities, menstrual cycles, drug use and current medications, were recorded.

All patients provided written informed consent for the study and bone biopsy, and the study protocol was reviewed and approved by the local ethics board and was registered in the Brazilian official trial registry (SISNEP) under number 3445–2010.

### Anthropometrics and clinical parameters

All participants underwent two anthropometric assessments thirty minutes after hemodialysis, and the arithmetic average was calculated. Body mass index (BMI = weight/height^2^) and percentage of body fat (%BF = 4.95/ST–4.50) were calculated as previously described [3].

The diagnosis of MetS was defined based on harmonization criteria as previously described [9]. These criteria included the following: 1) waist circumference >102 cm in men and >88 cm in women; 2) triglycerides >150 mg/dL and/or high-density lipoprotein (HDL) cholesterol <40 mg/dL in men or <50 mg/dL in women, or current use of a specific treatment for lipid abnormality; 3) blood pressure >130/85 mmHg or current use of hypertension therapy; and 4) fasting plasma glucose >100 mg/dL or specific treatment for hyperglycemia.

### Hormonal and biochemical analyses

Blood samples were collected on a dialysis-free day after an overnight fast. All analyses were performed at the same time and using the same standard kit. The following biochemical parameters were measured using colorimetric methods (*Vitros 950*, *Johnson & Johnson Chemistry Systems*, *USA*): phosphorus (2.5–4.5 mg/dL), albumin (1.0–6.0 g/dL), magnesium (1.6–2.4 mg/dL), glucose (>100 mg/dL), cholesterol (>200 mg/dL), HDL (<60 mg/dL) and triglycerides (>150 mg/dL). Chemiluminescent methods (Abbott Prism System, *Abbott Laboratories*, *USA*) were used to measure the following: intact parathyroid hormone (iPTH) (15–68.3 pg/mL), 25 (OH) vitamin D (30–60 ng/mL), insulin (<23 μUI/mL), thyroid-stimulating hormone (TSH), free tetra-iodo-thyroxine (T4), human follicle-stimulating hormone (FSH), and estradiol (E2) levels. We also assessed C-reactive protein (<1.0 mg/dL), total calcium (8.4–10.2 mg/d) (Radiometer ABL7000, *Radiometer America Inc*., *CA 92821*) and alkaline phosphatase (36–126 U/L) (heat inactivation, *Thermo Fisher Scientific Inc*., *USA*). The HOMA-IR (homeostasis model assessment-insulin resistance) was calculated using the following formula: glucose x insulin / 22.5. Female menopause was defined by laboratory values of FSH >40 mIU/mL, which indicated a 10-fold increase compared with levels in the follicular phase [10]. An enzyme-linked immunosorbent assay (ELISA) was used to measure fibroblast growth factor C-terminal 23 (c-FGF23, *Immutopic*, *San Clemente*, *CA*, *USA*), Scl (*Teco Medical Group San Diego*, *USA*), intact osteocalcin (OC, *Quidel Corporation San Diego*, *CA*, *USA*) and free serum leptin (*Teco Medical Group San Diego*, *CA*, *USA*). The value of leptin was adjusted based on gender and BMI, which is an approach used in previous population studies [11,12].

### Biopsy and bone histomorphometry

Bone biopsies were obtained according to a previously described technique [13]. In brief, a transiliac bone biopsy was performed using a Bordier trephine following a course of double labeling with tetracycline (20 mg/kg/day) for 3 days with a 10-day interval. The biopsy was performed 3–5 days after the last dose of tetracycline. Undecalcified specimens were fixed in 70% ethanol, dehydrated, and embedded in methyl methacrylate. The 5-μm-thick sections were cut using a Polycut S equipped with a tungsten carbide knife (Leica, Heidelberg, Germany), and some sections were stained with 0.1% toluidine blue (pH 6.4) for the analysis of static and bone marrow parameters. Unstained 10-μm-thick sections were obtained for the analysis of dynamic parameters by microscopy with ultraviolet light. All histomorphometric analyses were performed using a semi-automatic image analyzer and OsteoMeasure software (OsteoMetrics, Inc., Atlanta, GA, USA), at 125x magnification, and the full bone structure located between the two cortical areas was measured.

The following histomorphometric parameters were measured: the bone formation rate per unit of bone surface (BFR/BS, μm^3^/μm^2^/day) and mineralization lag time (MLT, days); bone volume (BV/TV, %); osteoid volume (OV/BV %); trabecular separation (Tb.Sp, /μm); trabecular number (Tb.N, /μm); and reabsorption surface (ES/BS %). All parameters were defined, measured, calculated and described as recommended by the guidelines of the American Society for Bone and Mineral Research detailed in Dempster et al. [14].

Bone histology was categorized according to the turnover-mineralization-volume (TMV) classification system described by Moe et al. [15] by determining the following parameters: bone turnover (T), defined as the BFR/BS; bone mineralization (M), defined by the MLT; and bone volume (V), defined by the BV/TV. Normal turnover was defined as BFR/BS values between -1 and +1 standard deviations (SD), high turnover was BFR/BS values greater than +1 SD, and low turnover was BFR/BS values lower than -1 SD of the mean reference values (men, n = 12: 0.13±0.07; women, n = 29: 0.07±0.03) [13]. Mineralization defect was defined as an MLT ≥50 days. Volume was defined as normal when the BV/TV values were between -1 and +1 SD, high when the BV/TV values were greater than +1 SD, and low when the BV/TV values were lower than -1 SD of the mean reference values (men, n = 47: 24.0±6.1; women, n = 40: 21.8±7.2) [16].

### Statistical analyses

Categorical variables are presented as percentages, and continuous variables are presented as the means±SD or median (minimum, maximum) when appropriate. The chi-squared test was used to evaluate differences in categorical variables. We also evaluated differences between groups with a t-test or a Mann-Whitney test. Spearman’s correlation was used to evaluate the biochemical components of MetS and bone histomorphometric variables. To explore the association of the TMV classification with MetS, we performed multiple regression analyses with robust standard errors with and without adjustment for potential confounding variables. The variables BFR/BS, MLT and BV/TV were used to define the TMV classification. For statistical analyses, SAS version 9.3 for Windows and GraphPad Prism 5 were used. Significance was defined as p<0.05.

## Results

Fifty-five patients (28 women, 27 men; 52 white) were evaluated. The causes of renal failure included hypertension (25.4%), diabetes mellitus (23.7%), glomerulonephritis (16.9%), other (10.2%) and unknown (23.7%). Because female dialysis patients often do not have regular menses, the diagnosis of menopause was based on FSH values [17,18], and menopause was present in 40% of this population. Females differed from males in many parameters, including a higher %BF (36.1 *vs*. 27.4, p<0.001), higher rates of turnover bone disease diagnoses (64.2% *vs*. 37.8%, p = 0.04), higher iPTH ([1,143 (28–3,000) *vs*. 358 (48–2,989) pg/mL, p = 0.02) and serum calcium levels (9.47 *vs*. 8.8 mg/dL, p = 0.02), higher total cholesterol (146.5 *vs*. 127.9 mg/dL, p = 0.03), higher OC (180.5 *vs*. 80 ng/mL) and lower fasting glucose levels (81 *vs*. 104 mg/dL, p = 0.03). Free leptin was higher in women; however, when the leptin values were normalized for age and BMI, there was no significant difference associated with gender. Phosphate binders (sevelamer in more than 80% of cases) were used by 94.5% of women and 92.6% of men, whereas calcitriol was taken by 53.5% of women and by 59.2% of men, with no significant difference between genders.

According to the TMV classification, the patients were evenly distributed between high and low bone turnover (51% and 49%, respectively). Bone volume was reduced in 31% of the patients, and bone mineralization was abnormal in 61.8% of the patients. There was no correlation between MLT and gender, age, dialysis duration, diabetes mellitus, the presence of MetS, %BF or calcitriol use.

As shown in Table 1, the hemodialysis patients were divided into groups according to normal or low BV/TV values. There were no differences in age, dialysis duration, diabetes, %BF or CKD-MBD-related treatment between these patients. Patients with normal BV/TV values exhibited higher BMI values (27.17 *vs*. 23.76, p = 0.007), as well as higher serum levels of phosphorus, c-FGF23 and fasting insulin. In addition, the prevalence of MetS among the patients with normal BV/TV was significantly higher than among the patients with low BV/TV values (73.8% *vs*. 41.1%, p = 0.02).

**Table 1:** Characteristics of the population distributed according to bone volume.

	NORMAL BV/TV (N = 38)	LOW BV/TV (N = 17)	p-value
Female	20 (52.6%)	08 (47.1%)	0.45
Age (years)	54.5 (49–62)	58 (44–65)	0.99
Dialysis duration	37 (27–72)	76 (36–108)	0.08
**MetS (%)**	**28 (73.7%)**	**7 (41.2%)**	0.02*
Diabetes (%)	13 (34.2%)	9 (52.9%)	0.19
**BMI kg/m**^**2**^	**27.2 (25–29.6)**	**23.7 (20.3–26.7)**	<0.01*
Body fat (%)	33.2 (30.4–37.1)	32.6 (22.6–36.5)	0.20
Serum creatinine (mg/dL)	8.7±2.4	9.1±2.5	0.64
Leptin/BMI	0.36 (0.29–0.81)	0.42 (0.23–0.80)	0.78
Calcium (mg/dL)	9.3 (8.7–9.8)	10 (9.5–10.2)	0.13
**Phosphorus (mg/dL)**	**5.8 (5.4–6.8)**	**5.1 (4.3–5.6)**	0.02*
iPTH (pg/dL)	971 (480–1306)	506 (320–1437)	0.62
Glucose (mg/dL)	85.5 (79–103)	106 (93–160)	0.06
Insulin (μUI/mL)	7.8 (6.3–8.8)	4.7 (3.3–8.6)	0.06
**HDL (mg/dL)**	**38.5 (35–44)**	**50 (40–65)**	0.01*
**c-FGF23 (RU/mL)**	**5,771 (3,083–10,320)**	**1,480 (488–4,177)**	<0.01*
Sclerostin (ng/mL)	1.02 (0.81–1.34)	1.16 (0.72–1.71)	0.85
Intact osteocalcin (ng/mL)	117.5 (47.3–193.5)	117.7 (74.4–230.6)	0.46
Phosphate binder Calcium based Sevelamer	36 (94.7%) 5 (13.1%) 31 (81.5%)	14 (82.3%) 1 (5.9%) 14 (82.3%)	0.39
Calcitriol	20 (52.6%)	13 (76.5%)	0.09

MetS: metabolic syndrome; BMI: body mass index; iPTH: intact parathyroid hormone; HDL: high-density lipoprotein; c-FGF23: C-terminal fibroblast growth factor 23. Values are expressed as the means±SD, median (min-max) or percentage.

*p<0.05.

**Fig 1:**
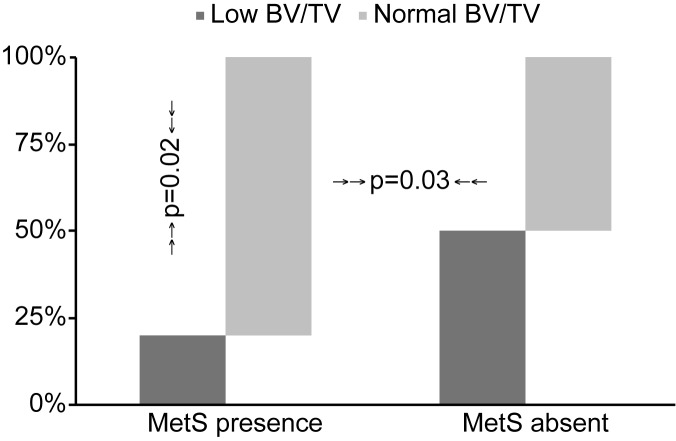
Percentage of low or normal BV/TV on biopsy according to presence or absence of MetS.

Regarding the diagnosis of MetS, as shown in Table 2, individuals with MetS were older and had a shorter dialysis duration than patients without MetS. There were no gender differences associated with MetS. As expected, MetS patients had higher BMI values, %BF, cholesterol, HOMA-IR, serum free leptin and a higher prevalence of diabetes. The BFR/BS, MLT and BV/TV values were not significantly different between the patients with and without MetS in the univariate analysis; however, the percentage of patients with normal BV/TV values was higher in the patients diagnosed with MetS (p = 0.02), as shown in Fig 1.

**Table 2:** Characteristics of the population distributed according to the diagnosis of metabolic syndrome.

	Without MetS (n = 20)	With MetS (n = 35)	p-value
Female	10 (50%)	18 (51.4%)	0.91
Age (years)	48.1±19.1	57.4±11.9	0.06
Diabetes (%)	3 (15%)	19 (54.3%)	<0.01*
Dialysis duration (mo)	114 (6–304)	36 (8–180)	<0.01*
BMI kg/m^2^	23.4±3.2	28.7±6.1	<0.01*
Body fat (%)	28.5 (6.7–36.3)	36.2 (10.6–46.6)	<0.01*
Calcium (mg/dL)	9.1±1.2	9.2±1.1	0.63
Phosphorus (mg/dL)	5.4±1.9	6.1±2.1	0.14
iPTH (pg/dL)	1,096 (130–2,989)	720 (43–1,643)	0.76
AP (U/L)	196 (53–734)	147 (73–978)	0.23
Albumin (g/dL)	3.8±0.5	3.7±0.4	0.82
CRP (mg/dL)	0.8 (0.5–23.3)	0.6 (0.5–21)	0.58
Glucose (mg/dL)	86 (68–155)	103 (72–356)	0.04*
Cholesterol (mg/dL)	126.7±34.7	143.5±29.6	0.06
LDL (mg/dL)	57.4±28.7	66.1±25.9	0.16
HDL (mg/dL)	42 (22–88)	40.5 (24–90)	0.13
Triglyceride (mg/dL)	92.1 (38–406.9)	155 (66–385)	<0.01*
Ferritin (ng/mL)	713.4 (194–6,274)	664.6 (60.2–4,420)	0.86
25-OH-VitD (ng/mL)	30.9 ±10.8	28.8±12.1	0.40
HOMA-IR	1.0 (0.53–3.3)	2.3 (0.3–24.8)	<0.01*
Insulin (μIU/mL)	5.5 (2.7–16.3)	8.4 (2.0–65.4)	<0.01*
Magnesium (mg/dL)	2.2 (1.4–2.9)	2.1 (1.6–2.9)	0.58
TSH (μIU/mL)	2.4 (0.2–9.1)	2.01 (0.59–16.97)	0.59
T4 (ng/mL)	1.1 (0.7–2.3)	0.97 (0.64–1.73)	0.33
Leptin (ng/mL)	6.5 (1.0–20.6)	17.05 (1–475.2)	<0.01*
Leptin/BMI	0.36 (0.29–0.81)	0.42 (0.23–0.80)	0.78
c-FGF23 (RU/mL)	5,315 (480–13,130)	3,552 (382–77,055)	0.06
Sclerostin (ng/mL)	0.9 (0.3–2.8)	1.75 (0.2–3.3)	0.30
Osteocalcin (ng/mL)	173.7 (12.3–556.2)	107.6 (9.3–414.1)	0.26
FSH (mIU/mL)	10.1 (3.1–150)	39.96 (0.24–150)	0.78
Estrogen (pg/mL)	24.5 (10–226)	26.5 (13–449)	0.32
BV/TV (%)	17.3±6.93	19.8±7.0	0.16
BFR/BS (μm^3^/μm^2^/day)	0.044 (0.0003–1.83)	0.049 (0.003–2.57)	0.66
MLT (days)	72,39 (5.99–100)	55.57 (8.41–100)	0.47

MetS: metabolic syndrome; BMI: body mass index; iPTH: intact parathyroid hormone; AP: alkaline phosphatase; CRP: C-reactive protein; LDL: low-density lipoprotein; HDL: high-density lipoprotein; 25-OH-VitD: 25 hydroxyvitamin d; HOMA-IR: homeostatic model assessment-insulin resistance; TSH: thyroid-stimulating hormone; T4: free tetra-iodo-thyroxine; c-FGF23: C-terminal fibroblast growth factor 23; FSH: follicle-stimulating hormone. Values are expressed as the means±SD, median (min-max) or percentage.

*p<0.05.

**Fig 2:**
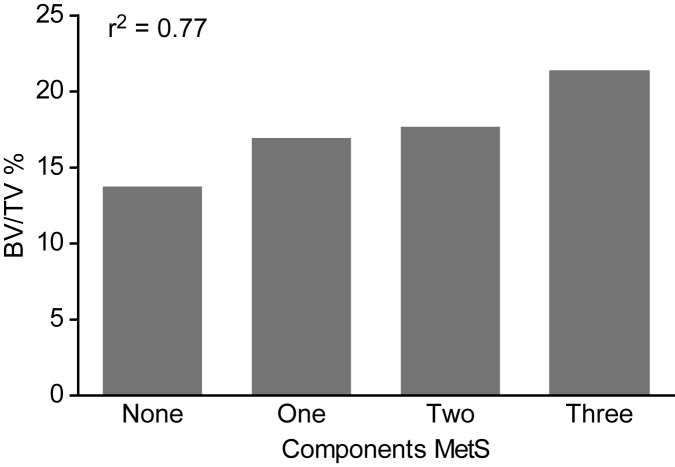
Correlation between MetS Components and BV/TV (r = 0.77, p<0.05).

Additional analyses were performed regarding the MetS components. Hypertension was present in 70.9% of patients. A waist circumference greater than 102 cm (men) or 88 cm (women) was observed in 61.8% of patients. Diabetes mellitus was detected in 47.2% and dyslipidemia in 52% of patients, and there were remarkably low HDL levels in all of the patients. Fig 2 shows that the number of MetS diagnostic criteria components correlated with the BV/TV value (r^2^ = 0.77, p<0.05). Conversely, BMI alone did not correlate with any of the bone histomorphometric parameters.

Additional correlations of the histomorphometric parameters and MetS components are shown in Table 3. BFR/BS, ES/BS, Tb.N and Tb.Sp correlated with cholesterol fractions (LDL and HDL). OV/BV and ES/BS showed negative correlations with glucose levels. BFR/BS correlated positively with iPTH and inversely with Scl. OV/BV and ES/BS also exhibited inverse correlations with Scl.

**Table 3:** Correlations between biochemical parameters and bone histomorphometry.

	BFR/BS	OV/BV	ES/BS	*TB*.*N*	*TB*.*SP*	*BV/TV*
Age		-0.39**		-0.28*	0.26*	
Glucose		-0.27*		-0.33**	0.30*	
Insulin			-0.29*			
LDL	0.41**		0.34**	0.28**	-0.26*	
HDL			0.31*		0.28*	-0.37**
Phosphorus	0.30*			0.30*	-0.29*	0.28*
iPTH	0.48**	0.47**	0.46**			
c-FGF23	0.36*			0.29*	-0.29*	0.31*
OC	0.52**	0.48**	0.57**			
Scl	-0.34**	-0.48**	-0.33**			

Spearman correlation coefficient (r^2^) *p<0.05, **p<0.01. BFR/BS: bone formation rate; BV/TV: trabecular bone volume; OV/BV: osteoid volume; ES/BS: eroded surface; Tb.N: trabecular number; Tb.Sp: trabecular separation; LDL: low-density lipoprotein; HDL: high-density lipoprotein; iPTH: *intact* parathyroid hormone; c-FGF23: C-terminal fibroblast growth factor 23; OC: osteocalcin; Scl: sclerostin.

Finally, a multivariate regression analysis was performed for dependent variables MLT, BFR/BS and BV/TV, and all models were adjusted for age. MLT was negatively associated with LDL cholesterol (r^2^ = 0.1492, p = 0.04), BFR/BS was negatively associated with Scl (r^2^ = 0.2156, p = 0.02), and BV/TV was negatively associated with age (r^2^ = 0.237, p = 0.03) but was positively associated with MetS (r^2^ = 0.237, p = 0.005). Table 4 shows the three models and their results.

**Table 4:** Multivariate analysis based on TMV classification.

	Turnover		Mineralization		Volume	
	β	p	β	p	β	p
Age	0.0003	0.546	-0,229	0,503	**-0.309**	0.039*
Dialysis duration					0.022	0.142
Leptin	0.0001	0.078			-0.165	0.272
LDL			**-0.341**	0.042*		
Scl	**0.023**	0.024*				
MetS	0.002	0.85			**0.515**	0.005*

LDL: low-density lipoprotein; Scl: sclerostin; MetS: metabolic syndrome. **β**: standardized coefficient beta.

*p<0.05.

Variables included in each model

Turnover model: age, leptin, c-FGF23, Scl, and MetS diagnosis.

Mineralization model: age, 25-OH-vitD, phosphorus, Scl, LDL, glucose, and BMI.

Volume model: age, MetS diagnosis, BMI, phosphorus, 25-OH-vitD, glucose, and c-FGF23.

## Discussion

In the present study, we examined the association between MetS and CKD-BMD. Patients diagnosed with MetS more frequently exhibited normal BV/TV than patients without MetS. Furthermore, several MetS components showed positive correlations with BV/TV. In contrast, high BMI without MetS diagnosis was not correlated with any of the bone histomorphometric parameters.

Many epidemiological studies have reported inconclusive and contradictory results regarding bone structural parameters and their relationship with MetS. Detrimental effects [19–22], positive effects [23–26] or a lack of influence [27] have been reported. Regarding MetS, no consensus has been reached concerning the influence of gender on bone mineral density or fracture risk. Studies in American [22,28] and Korean [20] populations have shown that MetS is an independent risk factor for low BMD only in men, but Chinese [21,29] studies found a higher prevalence of osteoporotic fractures only in women with MetS.

In our study, men and women showed different results. Women exhibited a higher prevalence of high turnover bone disease and higher total cholesterol, osteocalcin, calcium and %BF. Besides that in hemodialysis patients, bone and mineral disorders occur early in renal disease [30], and MetS is a risk factor for CKD [2,31]. However the percentage of patients with normal BV/TV was higher in individuals with MetS than in those without MetS, although gender, age, diabetes mellitus diagnosis and months on dialysis were not significantly different. In addition, the MetS group trended to be older (p = 0.06). A multivariate linear regression analysis adjusted for age, dialysis duration and serum leptin levels demonstrated a positive influence of MetS on BV/TV, which reinforced our findings.

Several diagnostic criteria components for MetS showed correlations with BV/TV, consistent with a previous study [19]. The pathophysiology of the correlation between MetS, its components and BMD in the general population is not completely understood but normal kidney function could be relevant [32], so these theories are not fully applicable to hemodialysis patients. There is strong evidence that a persistent inflammatory state, as occurs in hemodialysis patients, interferes with normal insulin signaling and inflammatory markers as interleukin 6 and C-reactive protein correlated with insulin resistance in clinical trials [33]. In addition, data from the Third National Health and Nutrition Examination Survey (NHANES III) linked 25-OH-vitD deficiency, another common feature of renal failure, with the prevalence of obesity and diabetes [34]. In our hemodialysis population, most patients were using calcitriol, particularly those with low BV/TV, and there are no clear data regarding the influence of the active form of vitamin D supplementation on insulin resistance and obesity.

A correlation of glucose levels with bone disease was previously observed in the Rotterdam Study [35] in which 3,458 participants were followed for 6.7 years. That study showed that glucose levels were positively associated with higher BMD in the femur neck. In our study, when patients with diabetes type 1 and type 2 were analyzed together, high glucose levels were associated with low Tb.N and higher Tb.Sp. Moreover, the group with normal BV/TV showed a strong tendency to have higher insulin and lower glucose levels than the low-BV/TV group (p = 0.06). As hyperglycemia becomes more evident, probably it is followed by advanced glycation end products. A direct anabolic of effect of hyperinsulinemia on the skeleton and independent of BMI has been previously reported [36], and this effect is probably mediated through the osteoblast-specific insulin-like growth factor 1 receptor [37].

We observed no association between total OC, an osteoblast-specific protein related to glycemic control [38], and serum glucose in our patients. This finding may be because total OC in renal osteodystrophy patients is high due to high bone turnover and is not able to reflect control over glucose metabolism.

Some studies have linked LDL cholesterol with osteoporosis in postmenopausal women, and fat consumption was identified as deleterious to bone in the Framingham Osteoporosis Study [39,40]. Several mechanisms could be involved, as the low absorption of dietary calcium and high prostaglandin synthesis stimulates the differentiation of osteoclast-associated lipid oxidation [41,42]. Another study that focused on primary hyperparathyroidism showed that low LDL was a predictor of vertebral fracture risk, regardless of age, weight, BMI, bone density and glomerular filtration rate [43]. In our sample, LDL cholesterol was positively correlated with BFR and eroded surface and was negatively associated with MLT in the regression models.

Obesity is related to improved BMD at some anatomical sites and has been linked to a reduction in hip fractures [3]. The rationale for this finding could be secondary to weight bearing and extragonadal estrogen synthesis in fat tissue [30,44]. A relationship between obesity and iPTH levels has previously been established in subjects with [45,46] or without CKD [47,48] and may be linked to 25-hydroxyvitamin D bioavailability, which is lower in obese people. In this study, we found higher iPTH levels associated with higher BMI and %BF, especially in women. However, the obesity indicated by BMI was not associated with the bone histomorphometric parameters.

In addition to MetS, studies in hemodialysis patients have shown that high levels of serum leptin are associated with adynamic bone disease [49]. Coen et al. [50] have shown reduced bone reabsorption associated with hyperleptinemia, particularly in men. We were not able to demonstrate any influence of leptin on the histological parameters evaluated.

Menopause was observed in 40% of the women in this sample population, and this number may be underestimated since we defined menopause based on FSH levels. Moreover, women had a higher prevalence of high turnover bone disease than men, based on BFR/BS and higher iPTH value. Parathyroid activity is influenced by sex steroid hormone, and long periods of unopposed estrogen exposure lead to stimulation of the gland. Since high turnover bone disease is more common in uremic women than men, female gender is associated with more aggressive histological findings and reduced control by calcitriol [51,52]. In our study, there were no differences in CKD-MBD treatment between genders; thus, this could not explain the significant iPTH difference between men and women. No gender differences were observed regarding bone volume or mineralization.

Of note, Scl, a Wnt/β-catenin pathway inhibitor capable of decreasing osteoblast activity associated with adynamic bone disease [53], showed a negative influence on BFR/BS in both genders.

This study had certain limitations, including a cross-sectional design, indirect methods for assessing body composition, considering only the trabecular bone and a small sample size. However, this study reveals novel data on the endocrine-metabolic influences on renal osteodystrophy, including histomorphometric analyses. Additionally, the complexity of the pathways involved in these conditions requires closely controlled experimental studies to address the relationship between MetS and bone physiology.

In conclusion, this study did not reveal MetS as a risk factor for low bone trabecular volume in hemodialysis patients. Instead, bone trabecular volume was positively influenced by the presence of MetS regardless of age, gender or dialysis duration.

## Supporting information

S1 Table MetS-osteodystrophy. Raw data of the study.
(XLS)
